# Supplementary material for: Antimycotic Effects of 11 Essential Oil Components and Their Combinations on 13 Food Spoilage Yeasts and Molds
Source: J Fungi (Basel). 2021 Oct 16;7(10):872. doi: 10.3390/jof7100872 (PMC8537543; doi:10.3390/jof7100872)
Supplement: Supplementary file 1 [file jof-07-00872-s001.zip › jof-1397788-supplementary.pdf]

Journal of Fungi

Supplementary Material to

Antimycotic Effects of 11 Essential Oil Components and Their Combinations on 13 Food Spoilage Yeasts and Molds

Laura Nißl, Florian Westhaeuser and Matthias Noll *

Coburg University of Applied Sciences and Arts, Institute for Bioanalysis, 96450 Coburg, Germany; lauraNissl@web.de (L.N.); florian.westhaeuser@hs-coburg.de (F.W.)
* Correspondence: matthias.noll@hs-coburg.de; Tel.: +49-9561-317645

ORCID: Laura Nissl: 0000-0003-3024-6565; **Matthias Noll:** 0000-0002-9981-5968

Supplementary Figure S1

CA / EOCs				0.125 MIC	0.25 MIC	0.5 MIC	1 MIC	2 MIC	0.125 MIC	0.25 MIC	0.5 MIC	1 MIC	2 MIC
	96	1	2	3	4	5	6	7	8	9	10	11	12
	A	PC	NC	MIC CA alone									
0.03125 MIC	B	MIC of EOC											
0.0625 MIC	C												
0.125 MIC	D												
0.25 MIC	E												
0.5 MIC	F												
1 MIC	G												
2 MIC	H												

Figure S1. Microtiter plate set-up for simultaneous MIC and FIC determination (modified from [1]). Grey shading indicate non-inoculated controls. (PC = positive control; NC = negative control)

1. Fratini F, Mancini S, Turchi B, Friscia E, Pistelli L, Giusti G, Cerri D (2017) A novel interpretation of the Fractional Inhibitory Concentration Index: The case *Origanum vulgare* L. and *Leptospermum scoparium* J. R. et G. Forst essential oils against *Staphylococcus aureus* strains. *Microbiol Res*. 195:11–17.
